# Higher pulse wave velocity in young adult offspring of mothers with type 1 diabetes: a case–control study

**DOI:** 10.1186/s12933-022-01612-7

**Published:** 2022-09-06

**Authors:** Cedric A. Korpijaakko, Mia D. Eriksson, Niko S. Wasenius, Miira M. Klemetti, Kari Teramo, Hannu Kautiainen, Johan G. Eriksson, Merja K. Laine

**Affiliations:** 1 Department of General Practice and Primary Health Care, University of Helsinki and Helsinki University Hospital, Helsinki, Finland; 2 Folkhälsan Research Center, Helsinki, Finland; 3 Department of Obstetrics and Gynecology, Helsinki University Hospital and University of Helsinki, Helsinki, Finland; 4 Department of Medical and Clinical Genetics, University of Helsinki, Helsinki, Finland; 5 Primary Health Care Unit, Kuopio University Hospital, Kuopio, Finland; 6 Yong Loo Lin School of Medicine, Human Potential Translational Research Programme and Department of Obstetrics and Gynecology, National University Singapore, Singapore, Singapore; 7 Agency for Science, Technology and Research (A*STAR), Singapore Institute for Clinical Sciences (SICS), Singapore, Singapore

**Keywords:** Cardiovascular disease, Offspring, Pregnancy, Pulse Wave Velocity, Type 1 diabetes

## Abstract

**Background:**

Offspring of mothers with type 1 diabetes have an increased risk for acquiring early onset cardiovascular disease (CVD). Arterial stiffness, measured as pulse wave velocity (PWV), is a non-invasive biomarker for CVD risk assessment. Our aim is to determine whether PWV is increased in young adult offspring of mothers with type 1 diabetes.

**Methods:**

This is a case–control study carried out in the hospital district of Helsinki and Uusimaa, Finland. 75 offspring of mothers with type 1 diabetes (cases) and 84 offspring of mothers without diabetes (controls), aged 18–23 years, were enrolled in this study. All participants attended clinical assessments, including questionnaires and laboratory tests. Carotid-femoral PWV (cfPWV), carotid-radial PWV (crPWV), and PWV ratio were measured from each participant using the Complior Analyse mechanotransducer (Alam Medical, France). Student’s t-test and chi-squared test were used to assess differences between the groups. Stata 17.0, StataCorp LP (College Station, TX, USA) statistical package was used for the analysis.

**Results:**

We did not observe any differences in conventional CVD risk factors: systolic blood pressure, LDL, Hb_A1c_, and smoking between cases and controls. We detected higher cfPWV in cases 6.5 (SD ± 1.2) m/s than in controls 6.2 (SD ± 0.7) m/s, p = 0.049, after adjustments for BMI, smoking, mean arterial pressure, height, and pulse rate was made. We did not observe any difference between cases and controls regarding crPWV or PWV ratio. Additionally, we detected no sex differences.

**Conclusions:**

We report a novel finding of signs of increased arterial stiffness already in young adult offspring of mothers with type 1 diabetes compared to matched offspring of mothers without diabetes. Our finding suggests that exposure to an adverse intrauterine environment of type 1 diabetes mothers may affect the vascular health of offspring already in young adulthood. Additional research within this topic is warranted.

## Introduction

The global burden of cardiovascular disease (CVD) is well-documented, accounting for over 30% of mortality in the United States alone [1]. To address the harmful effects of CVD, the United Nations included as one of their sustainable development goals to reduce preterm mortality from noncommunicable diseases by one third prior to 2030 [2]. Therefore, to achieve this long-term target, identifying new biomarkers for early cardiovascular risk assessment are needed.

The development of CVD is known to start already in childhood and young adolescence, simultaneously with the accumulation of CVD risk factors [3]. However, accumulating evidence suggests that an increased CVD risk could originate already in prenatal life. More specifically, the developmental origin of health and disease (DOHaD) hypothesis proposes that an abnormal intrauterine milieu may trigger fetal programming, effects that lead to elevated offspring risk of chronic diseases in adulthood [4, 5]. Indeed, there is evidence that maternal risk factors, such as diabetes and obesity, increase the risk for a poorer metabolic profile and early-onset CVD in the offspring [5–7].

In recent decades increased arterial stiffening has emerged as a potent biomarker for individual CVD risk evaluation. Stiffening of the large arteries initiates a complex pathophysiological process, resulting in elevated afterload and enhanced pulsatility in the peripheral tissues, which results in subclinical CVD damage [8, 9]. Arterial stiffening occurs naturally with aging [10, 11], however, the presence of traditional CVD risk factors, such as elevated blood pressure (BP), type 2 diabetes, dyslipidemia, and obesity, steepens the increase in vascular stiffness [10, 11]. Hence, arterial stiffness can be considered a distinct measure of biological aging within the vasculature [12].

It is possible to measure arterial stiffness through several non-invasive methods [13], and pulse wave velocity (PWV) has gained popularity. Carotid-femoral PWV (cfPWV) is accepted by the American Heart Association (AHA) as the gold-standard measurement for PWV [14]. Higher cfPWV, or increased arterial stiffness, has been associated with several traditional CVD risk factors [11, 15]. cfPWV has been shown to improve CVD event prediction and adding PWV to standard risk factors has been shown to reclassify individuals at intermediate risk [16]. It has previously been shown that the presence of CVD risk factors in adolescence, such as obesity and type 2 diabetes, accelerates vascular stiffening in comparison to healthy controls. This highlights that CVD risk factors affect vascular aging, thus also individual CVD risk already at a young age [17].

With the DOHaD hypothesis in mind, we wanted to assess whether CVD risk factors and arterial stiffness differed in young adult offspring of mothers with type 1 diabetes compared to matched offspring of mothers without diabetes. Our hypothesis was that PWV could serve as an early non-invasive biomarker for identifying early vascular changes in young adult offspring born to mothers with type 1 diabetes. The aim of this study was to determine whether increased PWV could be observed in young adult offspring of mothers with type 1 diabetes.

## Methods

This is a case–control study carried out in the Hospital District of Helsinki and Uusimaa, Finland. During the period between January 1st 1996 and January 31st 2000 all deliveries of type 1 diabetes pregnancies from the city of Helsinki and the province of Uusimaa were managed at the Department of Obstetrics and Gynecology, Helsinki University Hospital, Helsinki, Finland. All case participants, i.e., offspring of mothers with type 1 diabetes (n = 238), were delivered through elective caesarean section from a singleton pregnancy. The control participants (n = 476) consisted of the first or the second offspring of women without diabetes, delivered at different obstetrical departments across the Hospital District of Helsinki and Uusimaa, Finland. Data on birth of study participants were obtained from the Finnish Medical Birth Register (http://www.thl.fi/en/statistics/parturients) held by the Finnish Institute for Health and Welfare, Finland.

In 2019 all case and control participants were invited to a clinical study. The contact information of ten case participants and thirty-four control participants were not available. Eighty-one (36%) of the invited case participants (n = 228) attended, whereas 86 (20%) of the invited control participants (n = 442) attended. Four participants were excluded from the study population, as one control participant did not perform the standard 75 g 2 h-oral glucose tolerance test (OGTT), while three of the case participants were diagnosed with type 1 diabetes. In total, the study population consisted of 163 subjects.

The clinical study, including clinical assessment, laboratory tests, and questionnaires, was performed by trained study nurses.

Height was measured without shoes using a standardized measuring rod against the wall with an accuracy of 0.1 cm (SECA Telescopic measuring rod, SHZ, cm INT). A bioimpedance body composition device (InBody 3.0, Biospace, Seoul, South Korea) was used to measure body weight (kg) and fat mass (kg) of each participant in light indoor clothing without shoes and socks with an accuracy of 0.1 kg. BMI was calculated as body weight divided by height squared (kg/m^2^). Body fat percentage (%) was calculated as the ratio of fat mass (kg) divided by body weight (kg) multiplied by 100. BP was measured from each participant’s right arm with a standardized BP-monitor (Omron Intellisense M6 AC, Omron Healthcare Co. Ltd., Japan) in a sitting position after 15 min of rest, with a cuff size of 22 × 42 mm. The mean value of three BP measurements were documented, then inserted in the calculations of pulse pressure (PP = systolic BP—diastolic BP) and mean arterial pressure (MAP = diastolic BP + 1/3 × PP). The BP-monitor measured the pulse rate (beats per minute) simultaneously, which was documented.

PWV was measured from each participant after 10 h of fasting, in a silent and normal temperature room. The Complior Analyse device and software (Alam Medical, Vincennes, France) was used to assess PWV. The sensors were attached at the carotid-, radial-, and femoral arteries. The recordings were obtained by trained study nurses from each participant after 10 min of rest in the supine position. The mean value of two recordings was documented. PWV was calculated as the distance traveled (m) by the pulse wave between the two recording sites (carotid-femoral or carotid-radial) and divided by the pulse transit time (s) between the two sites. The PWV distance was measured as a direct line with a tape measure between the recording sites of the carotid artery and the femoral or radial artery. According to the general guidelines, this absolute distance was adjusted by a scaling factor of 0.8 [14]. We evaluated the cfPWV of each study participant which is a proxy measure of stiffness in the large elastic arteries (aorta), and the crPWV, which is a proxy measure of stiffness in the peripheral muscular arteries (brachial). Furthermore, we assessed the arterial stiffness gradient, or PWV ratio, by calculating the ratio of cfPWV to crPWV.

After 10 h of fasting, blood samples for assessment of HbA_1c_, fasting plasma glucose, LDL, HDL, total cholesterol, triglycerides, and high-sensitive C-reactive protein (hs-CRP) were collected from each participant. A 75 g 2-h oral glucose tolerance test (2 h-OGTT) was performed according to the World Health Organization’s (WHO) criteria with simultaneous assessment for insulin resistance by the homeostatic model assessment (HOMA-IR) [18].

Questionnaires were used to gather self-reported information regarding general health, chronic diseases, and smoking status.

Exercise was assessed using the Kuopio Ischaemic Heart Disease Risk Factor Study (KIHD) 12-month questionnaire of leisure-time physical activity (LTPA) [19]. Participants were asked to report their physical activity during the previous year (duration, number of occasions, intensity). Metabolic equivalent of task (MET)-values were assigned to each activity according to available data (1 MET = 3.5 ml O2/kg/min) [20]. The volume of LTPA was reported as MET-hours (MET-h) per week, which was calculated as a sum of product of each activities MET value, duration, and frequency.

This study was approved by the Ethics Committee of the Hospital District of Helsinki and Uusimaa (HUS/898/2017, 14 December 2017) and was conducted according to the ethical principles of the Helsinki Declaration. Prior to any clinical procedure written consent was provided by all study participants.

The descriptive statistics are presented as means with standard deviation (SD), and as counts with percentages. Group differences were evaluated using unpaired Student’s t-test, and chi-squared test, as appropriate. Relationships between PWV measurements in cases and controls, further divided by sex, was analyzed using two-way analysis of variance (ANOVA). In the case of violation of the assumptions (e.g., non-normality) for continuous variables, a bootstrap-type method or MonteCarlo p-values (small number of observations) for categorical variables was used. The normality of variables was evaluated graphically and by using the Shapiro–Wilk W-test. The Stata 17.0, StataCorp LP (College Station, TX, USA) statistical package was used for the analysis.

## Results

Table 1 shows the characteristics of the study population. The mean age of the cases was 20.5 (SD 1.6) years, whereas the mean age of the controls was 20.6 (SD 1.6) years.

**Table 1:** Clinical characteristics of study participants divided into offspring of mothers without diabetes (controls) and offspring of mothers with type 1 diabetes (cases)

	Controls n = 84	Cases n = 75	P-value
Age (years), mean (SD)	20.6 (1.6)	20.5 (1.6)	0.77
BMI (kg/m^2^), mean (SD)	24.1 (4.8)	24.5 (5.0)	0.64
Body fat percentage (%), mean (SD)			
Women	30.1 (9.2)	32.9 (7.7)	0.099
Men	18.4 (8.0)	19.3 (10.8)	0.72
BP (mmHg), mean (SD)			
Systolic	119 (11)	117 (12)	0.23
Diastolic	74 (7)	74 (9)	0.66
Comorbidity, n (%)			
Asthma	11 (13)	9 (12)	0.89
Atopy	7 (8)	10 (14)	0.28
Cardiovascular disease	4 (5)	5 (7)	0.73
Inflammatory bowel disease	3 (4)	7 (10)	0.19
Mental and behavioral diseases	18 (21)	11 (15)	0.31
Neurologic diseases	10 (12)	12 (16)	0.41
Psoriasis	1 (1)	3 (4)	0.34
Rheumatic diseases	2 (2)	0 (0)	0.50
Fasting insulin (mU/L), mean (SD)	10.6 (6.5)	11.2 (9.1)	0.65
Glucose (mmol/l), mean (SD)			
0-h	5.40 (0.43)	5.39 (0.41)	0.84
2-h	5.62 (1.43)	5.85 (1.64)	0.34
HbA_1c_ (mmol/mol; %), mean (SD)	33 (2); 5.13 (0.20)	33 (3); 5.15 (0.25)	0.54
High sensitivity C-reactive protein (mg/L), mean (SD)	1.4 (1.9)	2.0 (4.9)	0.56
LTPA (MET-h/week), median (IQR)	24.3 (11.6, 41.3)	24.1 (13.8, 34.7)	0.89
Mean arterial pressure (mmHg), mean (SD)	89 (7)	88 (9)	0.41
Pulse pressure (mmHg), mean (SD)	45 (8)	43 (9)	0.26
Pulse rate (bpm), mean (SD)	76 (12)	75 (12)	0.74
Smoking, n (%)			
No	62 (74)	60 (80)	0.50
Occasionally	12 (14)	10 (13)	
Continuously	10 (12)	5 (7)	
Total cholesterol (mmol/L), mean (SD)	4.31 (0.65)	4.40 (0.75)	0.41
LDL (mmol/L), mean (SD)	2.56 (0.66)	2.59 (0.70)	0.73
HDL (mmol/L), mean (SD)			
Women	1.65 (0.38)	1.68 (0.39)	0.70
Men	1.38 (0.30)	1.45 (0.32)	0.37
Triglycerides (mmol/l), mean (SD)			
Women	0.97 (0.48)	0.94 (0.62)	0.81
Men	0.94 (0.35)	0.85 (0.34)	0.39
Women, n (%)	55 (65)	50 (67)	0.87

*LTPA* leisure-time physical activity, *MET-h* Metabolic equivalent of task-hours, *SD* standard deviation

We did not observe any differences between cases and controls in BMI, systolic BP, diastolic BP, or LTPA.

**Fig. 1:**
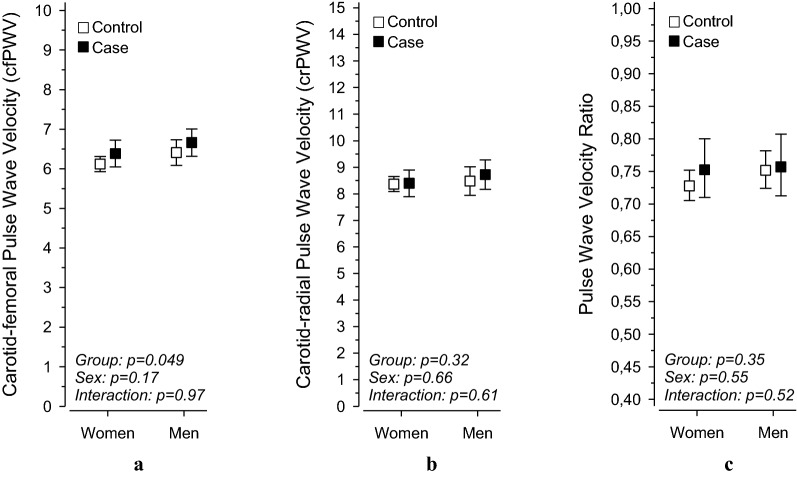
PWV measurements in offspring of mothers with type 1 diabetes (cases) and offspring of mothers without diabetes (controls), further divided by sex and type of PWV measured; cfPWV (**a**); crPWV (**b**); PWV ratio (**c**). Adjusted for: BMI, smoking, mean arterial pressure, height, and pulse. *cfPWV* carotid-femoral pulse wave velocity, *crPWV* carotid-radial pulse wave velocity, *PWV ratio* cfPWV/crPWV

Figure 1 shows the different PWV measurements in cases and controls, further divided by sex. The cfPWV values were higher in adult offspring of mothers with type 1 diabetes [6.5 (SD ± 1.2) m/s] than in adult offspring of mothers without diabetes [6.2 (SD ± 0.7)m/s, p = 0.049], after adjustments for BMI, smoking, height, MAP, and pulse rate. We did not find any differences between the cases and controls for crPWV or for PWV ratio, further adjustments did not change the results. We did not observe any significant differences between the sexes for cfPWV, crPWV, or for PWV ratio.

## Discussion

We report a novel and interesting finding that offspring of mothers with type 1 diabetes have increased arterial stiffness when compared to offspring of mothers without diabetes already in young adulthood. This observation would suggest that exposure to abnormal intrauterine environment—in this case maternal type 1 diabetes—during fetal development may affect vascular health in offspring already in young adulthood.

Previous studies have shown that adult offspring of mothers with diabetes have an increased risk of acquiring early onset CVD. In a Danish population-based cohort study, Yu et al. reported that during a 40-year follow-up, offspring of mothers with pregestational diabetes had a hazard ratio (HR) of 1.29 to develop early onset CVD [6]. Although the exact mechanism remains unknown, the earliest signs of CVD could originate already in the developing fetus, possibly mediated through epigenetic mechanisms [21]. Murray et al. reported that maternal risk factors during pregnancy could induce DNA methylation in the offspring’s genome at birth, which in turn, was found to be associated with enhanced arterial stiffening after a follow-up of 8–9 years [22]. In addition, offspring of mothers with pregestational diabetes have been observed to have an increased risk for pathological ventricular hypertrophy already at birth [23]. Albeit there might be a relationship between individuals exposed to maternal type 1 diabetes in utero and the risk of CVD, yet surprisingly, we could not find any signs of clinical CVD in young adulthood. One possible explanation might be related to the young age of our study participants. The vascular changes that occur in order to develop CVD most often consist of a several decades-long process, and naturally, the incidence of pathognomical abnormalities of vascular disease are much rarer in young adults [1]. This would require more invasive measures to identify. Also, the role of epigenetics in the transmission of CVD risk heritage may be one of the mechanisms involved, however, as there is still a gap in the understanding of epigenetics, its role in the development of CVD remain incomplete [24].

In 2010 the European Society of Cardiology (ESC) established reference values for cfPWV measurements per age decade and for different BP levels; for instance, in under 30-year-olds with optimal BP-levels (less than 120/80 mmHg), the 90th percentile for cfPWV was under 7.0 m/s [25]. In addition, an expert consensus statement proposed that a cut-off value of 10 m/s for cfPWV to be used in the prediction of CVD events [26]. This could be interpreted to suggest that subclinical organ damage is not present in our study population, although the mean cfPWV value that we observed in offspring of mothers with type 1 diabetes was higher than the mean cfPWV observed in offspring of mothers without diabetes. However, due to the lack of published studies measuring PWV in adults born to mothers with pregestational diabetes, it is difficult to interpret our results in a generalized context. In 2020, Yuan et al. assessed cfPWV in six-year-olds born to mothers with gestational diabetes, however, they observed increased stiffness only in male offspring of mothers with gestational diabetes for cfPWV [27]. When comparing our results to study populations with similar ethnic and age profiles, e.g. Petterson-Pablo et al. assessed vascular status in healthy Swedish young adults (mean age 22 ± 2.0 years) [28]. The mean cfPWV value in both male participants (5.6 ± 0.86 m/s) and female participants (5.2 ± 0.69 m/s) was lower than the value we observed in our study participants [28]. Longitudinal studies have reported that increased arterial stiffness could precede the development of hypertension and diabetes, supposedly being the first change to occur in the development of CVD [29, 30]. Taken together, our results suggest that young adult offspring of mothers with type 1 diabetes have subtle vascular stiffening, which may convert into clinical CVD later in life.

There is mounting evidence to support findings that offspring of mothers with type 1 diabetes have an increased risk for developing obesity, abnormal glucose regulation, and a poorer metabolic profile than offspring born to mothers without diabetes [31, 32]. More worryingly, Lindsay et al. reported that the increased risk for overweight and obesity in offspring of type 1 diabetes mothers can be observed already in childhood [33]. The presence of CVD risk factors in childhood, such as adiposity, hypertension, and dyslipidemia, have a tendency to track into adulthood, consequently affecting CVD risk already at younger ages [34]. Indeed, the Cardiovascular Risk in Young Finns-study reported that the presence of risk factors, such as obesity and type 2 diabetes, severely accelerates vascular stiffening already in childhood, and that only the reduction of CVD risk factors or weight loss predicted the reduction in arterial stiffness in adulthood [35]. Yet, surprisingly, we were unable to find any significant differences in the traditional CVD risk factors between cases and controls in this study. One possible explanation might derive from a healthier lifestyle among offspring of mothers with type 1 diabetes. We believe that the implementation of lifestyle counseling in the Finnish society for all type 1 diabetes patients might have benefitted the habitual patterns within these families. It was recently noticed that offspring of mothers with better cardiovascular health developed later CVD in comparison to offspring of mothers with poorer cardiovascular health [36]. Consequently, a healthy and favorable lifestyle during childhood could have suppressed any clinical differences that might have otherwise occurred between the two study groups.

Further, chronic inflammation is known to accelerate the formation of atherosclerotic lesions within the vasculature and is present early in the development of CVD [37, 38]. As a marker of chronic inflammation, we assessed hs-CRP in our study population, however, this result remained non-significant between the groups.

For a comprehensive arterial stiffness analysis, we included crPWV and PWV ratio assessment in our study participants. The PWV ratio (cfPWV/crPWV) derives from changes that occur with aging, as central arterial stiffness increases, peripheral arterial stiffness remains or decreases [39]. This results in an inversed arterial stiffness gradient, which has shown to provide an alternative method for predicting CVD outcomes [40]. The authors of the Framingham Heart Study addressed this question in a general population setting, concluding that PWV ratio did not improve CVD prediction in comparison to cfPWV, and that cfPWV should be used as the standard measure of arterial stiffness [41]. In the present study, we could not observe any significant differences between the groups for crPWV or for PWV ratio. Additionally, the PWV ratio is under 1 in both cases and controls, suggesting that the arterial stiffness gradient is not inversed. Although our study is limited to a small population size, our results indicate that a normal arterial stiffness gradient, or PWV ratio under 1, might not be a suitable method for assessing early subclinical CVD changes in younger age groups.

It is widely acknowledged that physical activity has beneficial effects in reducing the risk for acquiring non-communicable diseases [42]. Hence, it is unsurprising, that increased physical activity reduces arterial stiffness [43, 44]. We assessed physical activity as the volume of LTPA in our study participants, however, we were unable to find any differences (p-value 0.89) between the cases (24.1 MET-h/week) and controls (24.3 MET-h/week). Wahid et al. conducted a meta-analysis investigating the relationship between physical activity with CVD and diabetes [45]. In comparison to inactive behaviour, the meta-analysis found that medium physical activity (defined as 11.5–29.5 MET-h/week) almost reduced the relative risk of incident type 2 diabetes by 30 per cent and incident CVD by 20 per cent [45]. The observed similarities in total LTPA among our study participants could further strengthen that a healthy lifestyle in young adult offspring of type 1 diabetes mothers may have suppressed any clinical CVD differences.

This study has several strengths. To the best of our knowledge, this is the first study conducted to assess whether early subclinical CVD, measured as PWV, can be found in young adult offspring of mothers with type 1 diabetes. Furthermore, we have made a thorough evaluation of traditional CVD risk factors in every study participant. Also, this study population is unique and homogeneous, as the participants consist of same aged, normotensive, normoglycemic, and normal weight participants, thus minimizing other confounding factors on PWV. In addition to cfPWV measurements, we added crPWV and PWV ratio in our analysis, for comprehensive assessment of arterial stiffening.

Some limitations also need to be taken into account when considering our study results. First of all, the cross-sectional study design prevents further comments on causality. All study participants were with Finnish background, which limits the generalization of the results.

The overall participation rate was low, i.e., only 36% of the invited case participants attended. This might have affected the observed differences between the study groups, excluding people with more health problems, and consequently, leading to overrepresentation of healthy participants. Thus, the possibility of selection bias cannot be fully excluded. The role of epigenetics in the DOHaD hypothesis seems feasible, however, we could not assess its potential role in our study population. Lastly, we did not assess other subclinical CVD risk tools, such as carotid intima-media thickness, which could provide additional information on subclinical vascular changes.

We report a novel finding of increased arterial stiffness in young adult offspring of mothers with type 1 diabetes compared to offspring of mothers without diabetes. We could not observe any differences in traditional CVD risk factors between cases and controls, thus suggesting that arterial stiffening might be one of the early subclinical CVD changes to occur. However, since previous studies suggest that offspring of mothers with type 1 diabetes are more prone to a unfavorable metabolic profile than offspring of mothers without diabetes, from a preventive perspective it is of vital importance to encourage these individuals to maintain a sustainable and healthy lifestyle, reducing the cumulative exposure of CVD risk factors on the vascular wall, which would further increase arterial stiffening and the burden of CVD. Follow-up studies are warranted.

## Data Availability

The datasets generated and/or analysed during the current study are not publicly available due to legal reasons.

## Abbreviations

cfPWV: Carotid-femoral pulse wave velocity
crPWV: Carotid-radial pulse wave velocity
DOHaD: Developmental Origin of Health and Disease
LTPA: Leisure-time physical activity
MET: Metabolic equivalent of task
PWV: Pulse wave velocity
